# Prominent gamma band activity during visual motion perception in early-stage Alzheimer’s disease

**DOI:** 10.1371/journal.pone.0266693

**Published:** 2022-04-18

**Authors:** Nobushige Naito, Tetsu Hirosawa, Makoto Tsubomoto, Yoshiaki Miyagishi, Mitsuru Kikuchi

**Affiliations:** 1 Department of Psychiatry & Behavioral Science, Kanazawa University, Kanazawa, Japan; 2 Research Center for Child Mental Development, Kanazawa University, Kanazawa, Japan; Hamamatsu University School of Medicine, JAPAN

## Abstract

**Introduction:**

Alzheimer’s disease (AD) affects multiple neural pathways and regions, resulting in various visual impairments such as motion perception. Generally, gamma-band activities during visual motion perception have been thought to reflect ongoing cognitive processes. Nevertheless, few studies have specifically examined induced gamma band activity during visual motion perception in AD patients. Therefore, after performing magnetoencephalography (MEG) recording during apparent motion (AM) stimulation for the left hemi-visual field in patients diagnosed as having AD in the early stage, we compared the results with findings of cognitive performance.

**Methods:**

Seventeen AD patients in the early stage and 17 controls matched for age, sex, and educational attainment participated in this study. For each participant, memory performance was assessed with the Mini-Mental State Examination (MMSE) and the Wechsler Memory Scale-Revised (WMS-R). For MEG analysis, we examined power changes induced in a higher frequency range (20–100 Hz) after AM stimuli.

**Results:**

The power of induced gamma band activities was significantly higher in AD patients. The power of induced gamma band activities was associated with higher performance on both MMSE and WMS-R tests for attention and concentration in AD patients.

**Conclusions:**

Given that neuronal dysfunction in AD is associated with excitotoxic neurodegeneration, and given that subsequent development of compensatory inhibitory mechanisms also contributes to pathology in AD patients, elevated gamma band oscillations might reflect an imbalance of inhibitory and excitatory activity in AD patients. Moreover, positive correlation between induced gamma activity and cognitive performance might signify a compensating mechanism of inhibitory neurons which preserve the pyramidal neuron from excitotoxicity in a posterior association area.

## Introduction

Alzheimer disease (AD) is the most prevalent form of dementia. Aging is the most important risk factor for AD. As an unavoidable consequence of longer life span during recent decades, increasing numbers of people reach ages at which AD commonly occurs. Neuronal dysfunction associated with excitotoxic neurodegeneration has been reported to occur with Alzheimer disease (AD) [1–5]. Clinically, this mechanism seems plausible because of increased risk of seizure activity in AD [6]. Results of a recent human immunohistochemical study suggest that neuronal overexcitation and subsequent development of compensatory inhibitory mechanisms contribute to pathology in AD [7]. Transgenic mice studies also elucidated this compensatory inhibitory mechanism induced by amyloid-β [8, 9]. Although it remains unclear whether these neuronal imbalances of inhibition–excitation are a cause or effect of pathological degeneration, these imbalances must play an important role in the pathological progress of AD patients.

Gamma band activity detected by electroencephalography (EEG) or magnetoencephalography (MEG) is a candidate non-invasive index that accounts for inhibitory–excitatory neuronal dynamic alteration in the human brain. Inhibitory interneuron networks are well established as having a prominent role in the generation of gamma-band activity. Activation of these interneuron networks is attributable to excitatory driving from pyramidal cells [10]. Nevertheless, only a few studies of AD patients have specifically examined locally induced gamma band oscillations that are not time-locked or phase-locked to a stimulus, but which are elevated during meaningful information processing [11]. One study demonstrated that the AD patients had higher induced gamma band power than control subjects had. These powers were positively correlated with cognitive performance [12]. This finding suggests that local cortical activities are preserved or are rather higher in AD, which might reflect hyper-excitability of the cortex or a compensation mechanism by inhibitory interneurons, which counters hyper-excitability in AD.

Presuming that the hyper-excitability of the cortex and compensatory inhibitory mechanisms contribute to higher induced gamma activity in AD patients, more specific brain activation particularly addressing brain local regions that are commonly vulnerable in AD can elevate the diagnostic sensitivity of gamma band activity. For this study, we employ apparent motion (AM) visual stimulation. The AM stimulation consists of static images that are presented successively with a spatial shift. It provides simple information about object motion. Using this stimulus, earlier MEG studies of healthy participants revealed activated cortical origins that were estimated around temporo-occipital, occipital or parietal areas [13]. It is noteworthy that amyloid deposition and metabolic reduction in AD patients was reported in these areas [14]. Nevertheless, no report describes a study investigating visual motion-induced brain gamma band activities and their relation to cognitive dysfunction in patients with early AD.

The purpose of the current study is to evaluate the diagnostic sensitivity of locally induced gamma band MEG activities in discriminating AD patients from healthy controls. Because neuronal overexcitation and the compensatory inhibitory mechanisms seem to contribute to pathology in AD, we expected that regionally induced gamma band activity would be aberrant in early-stage AD patients.

## Materials and methods

### Participants

The characteristics of AD group and control group participants are presented in Table 1. The AD patient group consisted of nine women and eight men who were referred to the psychiatric outpatient clinic of Kanazawa University Hospital or Kaga Kokoro Hospital. The patients fulfilled the National Institute of Neurological and Communicative Diseases and Stroke/Alzheimer’s disease and Related Disorders Association (NINCDS-ADRDA) work group criteria for probable AD [15]. Neurological, serological, and MRI studies of these patients were performed to eliminate any other medical condition potentially causing dementia. The period from the time the symptoms were noticed by the family was defined as the duration of illness. No patient was receiving any medication acting upon the central nervous system (i.e. antipsychotic, anticholinergic, antidepressant, anticonvulsant, benzodiazepine, cerebral metabolic activator, or cerebral vasodilator) except donepezil hydrochloride (9 patients were receiving 5 mg of donepezil hydrochloride per day). Each patient was assessed with the functional assessment stages (FAST) [16] (CDR) [17], and a Japanese version of the Mini-Mental State Examination (MMSE) [18]. The Wechsler Memory Scale-Revised (WMS-R) [19] was used to evaluate details of memory performance. The control group, which consisted of 17 healthy volunteers, was not significantly different from the AD group in age or gender. Control participants had no personal or family history of psychiatric or neurological disease. All were functioning normally and independently in daily life. None of their WMS-R subscores was below the 1.5 standard deviation of the normal range. All participants had normal color vision, visual acuity, pupil reaction, and visual fields. All participants agreed to participate in the study with full knowledge of the experiment characteristics of the research. Written informed consent was obtained from all participants before their enrollment. The Ethics Committees of Kanazawa University Hospital approved this study.

**Table 1:** Characteristics of all participants.

Group	Healthy controls	Alzheimer disease	*T* value
Total number	17	17	
Male/Female	9/8	9/8	
Age (range) years	69.6 (61–77)	72.0 (63–80)	1.36
Education (range) years	12.4 (6–16)	11.2 (6–16)	0.28
Duration of illness (range) years	–	1.6 (1–5)	
CDR	–	0.94 (0.5–2)	
FAST	–	3.6 (3–4)	
MMSE score	28.5 (27–30)	22.4 (14–28)	6.71 *
WMS-R indexes			
Attention and Concentration Index (range) ^a^	108.1 (89–125)	87.1 (53–109)	4.28 *
General Memory Index (range) ^b^	101.2 (84–122)	65.6 (50–93)	8.40 *
Verbal Memory Index (range)	100.4 (84–121)	70.3 (53–97)	7.82 *
Visual Memory Index (range)	102.7 (80–125)	67.5 (50–100)	7.44 *
Delayed Recall Index (range) ^c^	99.9 (87–130)	61.4 (50–81)	9.82 *

CDR, Clinical Dementia Rating; FAST, Function Assessment Staging; MMSE, Mini-Mental State Examination; WMS-R, Wechsler Memory Scale–Revised

^a^ Immediate memory (retention of information in immediate awareness).^b^ Capacity to acquire and recall information over brief time periods.

^c^ Ability to recall information after 30 min of intervening activity. Significance was inferred from results of un-paired *t*-tests.

* *P* < 0.05.

### Visual stimuli

**Fig 1:**
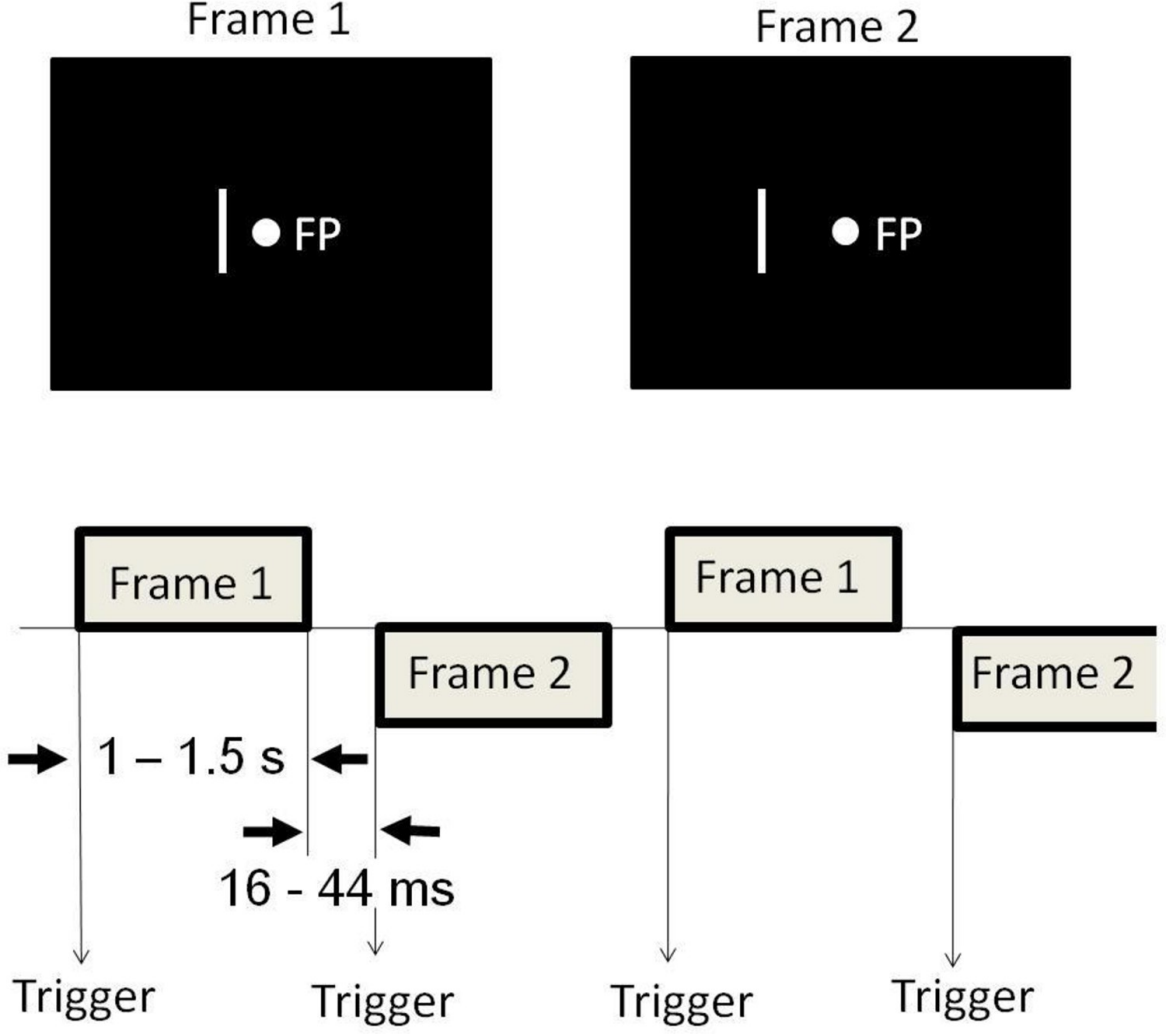
Schematic illustrations of the visual stimuli and a diagram showing the MEG acquisitions. Visual stimuli used to induce apparent motion (AM) were created using two frames: Frame 1 had a fixation point (FP) and a bar offset 1 degree (visual angle) from that point; Frame 2 had an FP at the same location as in Frame 1 and a bar offset 2 degrees from that point. Frames 1 and 2 were presented alternately for 1 to 1.5 s with an interstimulus interval of 16–44 ms. Trigger pulses for the averaging of magnetic responses occurred whenever the frame changed.

A participant lay supine on the bed, facing a tilted white screen with 24 × 16 cm that was fixed above the bed in a dark, magnetically shielded room (Daido Steel Co., Ltd., Nagoya, Japan) in which the MEG apparatus was set. Using a video projector (PG-B10S; Sharp Corp., Japan) with a refresh rate of 60 Hz, a computer placed outside the magnetically shielded room projected a picture through a small window of the wall of the shielded room, onto the screen above the head position of the bed. The distance from the participant’s nasion to the center of the screen was about 30 cm. Therefore, the visual angle of the picture that was projected on the screen was about 42 × 34 degrees. Visual stimuli were generated by software “Presentation” (Neurobehavioral Systems Inc., Albany, USA). Diagrams of the experiment procedures are shown in Fig 1. We used a visual AM stimulus similar to that used for an earlier study [13]. The visual stimulus consisted of two frames. Each frame had a green spot (0.27 degrees visual angle diameter) as the fixation point and a white vertical bar (subtended 0.2 × 3.9 degrees) on the left of the fixation point. The bar was located at a 1.0 degree and a 2.0 degree offset from the fixation point, respectively for Frames 1 and 2. The frames were presented alternately so that participants would see a horizontal to-and-fro motion of the bar between the places where the bars were presented. The duration of the presentation of each frame was randomized between 1 and 1.5 s to eliminate averaging late activities which might be evoked by the preceding stimulation and to prevent the participant’s expectation and adaptation to the stimuli [20]. The interframe interval was 16–44 ms because of synchronization to the monitor frame flyback time. The respective measured luminance of the bar and the screen background were 38.5 and 4.3 lux.

### Acquisition and data analysis of MEG evoked responses

MEGs were recorded with a 160-channel, whole-head coaxial gradiometer MEG system (PQ 1160C; Yokogawa Electric Corp., Kanazawa, Japan) in the magnetically shielded room installed at the MEG Center of Yokogawa Electric Corp. Each gradiometer had 15.5-mm-diameter pick-up coils. The coils were separated by 25 mm. The instrument sensitivity was 3 fT/Hz^0.5^ or better. MEG was sampled at 5000 Hz per channel (band pass 0.1–3000 Hz). The participant’s head was placed in a whole-head dewar, in which 160 magnetic sensors had been arranged concentrically.

We determined the head position within the helmet by measuring the magnetic fields after passing currents through coils that had been attached at five locations of the head surface as fiduciary points with respect to the landmarks (nasion and pre-auricular points). A 3-D head coordinate system was applied to express the location and orientation of equivalent current dipoles (ECDs).

**Fig 2:**
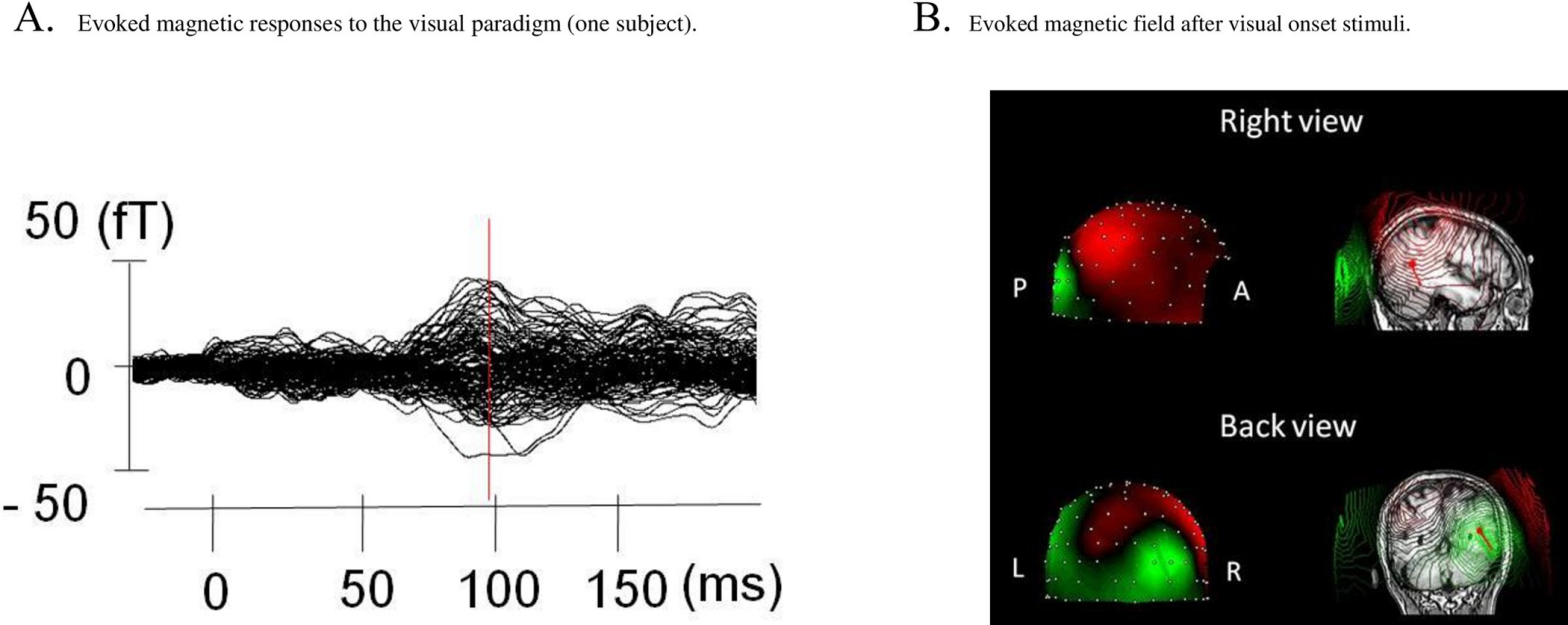
A, Representative example of the magnetic responses to the visual stimuli obtained from one participant. MEG waveforms (160 channels) are overlaid at the corrected baseline. Isocontour maps of the magnetic field (strength denoted by color, varying from green (flux-in) to red (flux-out)) at the response peak and sensor locations (white dots) are shown. B, Isocontour maps of the magnetic field after apparent motion visual stimuli. A, anterior direction; P, posterior direction; L, left; R, right.

The recordings (sampled at 5000 Hz) were resampled at 500 Hz after a low pass filter of 200 Hz was applied. Then we made segmentation of these time series around the onset of AM (- 300 to 700 ms) and after the baseline correction (-50 to 0 ms). At least 200 segments were averaged for each 160 magnetic sensor. Segments contaminated by noise with large amplitude exceeding ± 4 pT were removed from the analysis. As shown in Fig 2, dominant activities were found around 100 ms after the onset of AM in this study. The single ECD model [21] was used to estimate the “center of gravity” of the current sources in the activated cerebral cortex that was detected by at least 20 sensors evoked by the visual stimuli. We accepted estimated ECDs only when they fulfilled the following criteria: First, during the response that we specifically examined, the location of estimated dipoles with the single ECD model was stabilized within ±8 mm of each coordinate. Second, the correlation between the measured and expected magnetic fields remained at 0.9 or more during the period 10 ms around the peak of the response. When ECDs were estimated in bilateral hemispheres, we adopted ECD from either hemisphere in which correlation between the measured and expected magnetic fields was higher.

### Analysis of induced MEG activity

**Fig 3:**
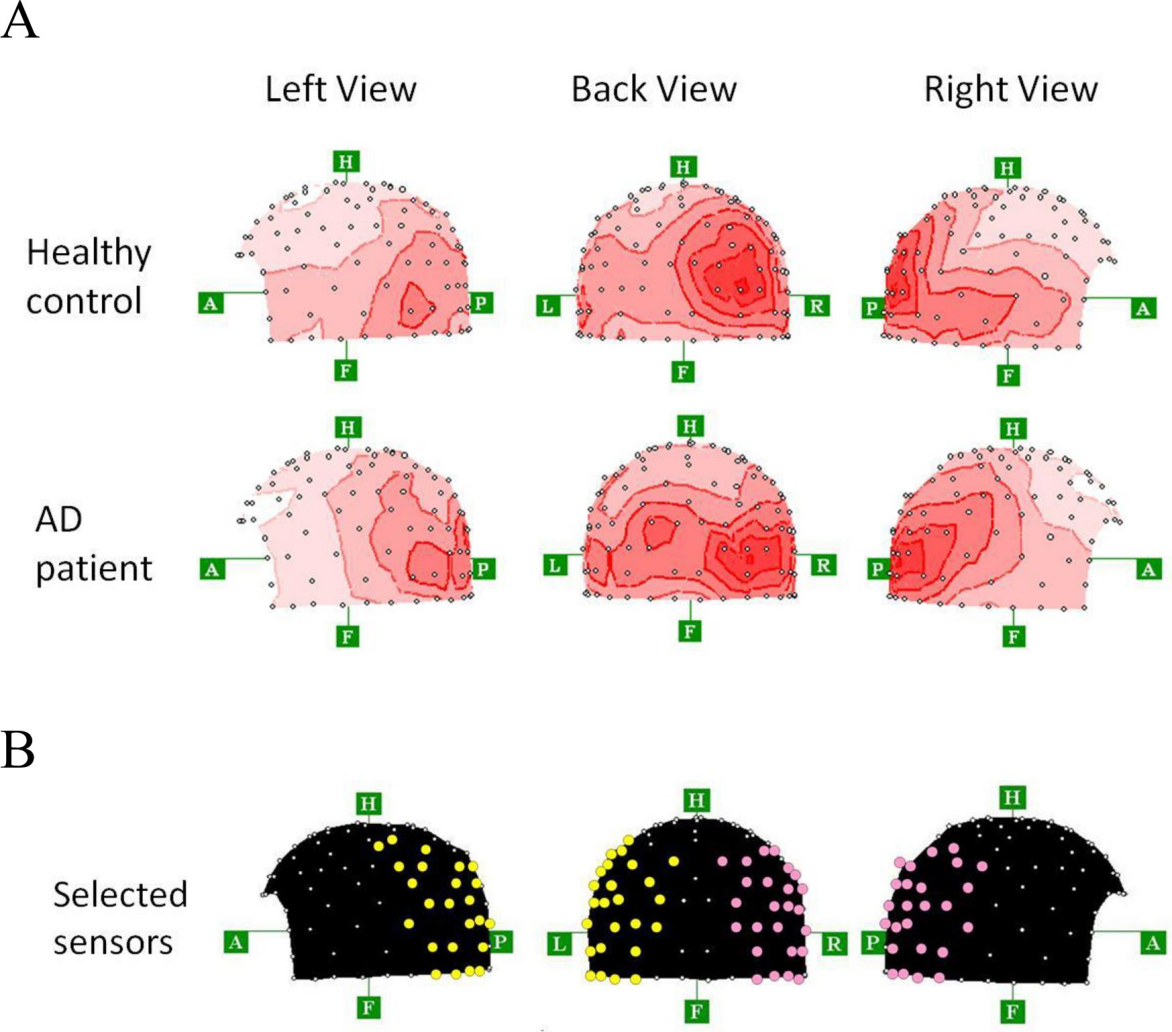
A, Grand averaged topographies from rectified averaged data at the peak of RMS (using 160 sensors) after AM stimuli for the healthy controls (*n* = 17) and AD patients (*n* = 17). B, Location of 25 sensors (red circles for right hemisphere, yellow circles for left hemisphere), which seemed to cover the magnetic response to the apparent motion stimuli for each hemisphere. These sensors were located mainly at the posterior areas. A, anterior direction; P, posterior direction; F, foot direction; H, head direction; L, left direction; R, right direction.

Aside from estimating ECDs after AM stimulation, we attempted to analyze the induced MEG activities. To examine AM-related activities specifically, we selected the 25 fixed sensors for both right and left hemispheres (Fig 3B), which seemed to contain the magnetic response to the AM stimuli. Selection of the 25 fixed sensors was done using the following procedures. First, we calculated the root mean square (RMS) of the averaged MEG traces obtained from 160 channels. Then we made grand averaged topographies from rectified averaged data at the peak of RMS (using 160 sensors) after AM stimuli (Fig 3A). As shown in Fig 3A, no apparent difference was found in these topographical features between healthy controls and AD patients. Then, according to these grand averaged topographies, we selected the 25 fixed sensors for each hemisphere which seemed to include the magnetic response to the AM stimuli (Fig 3B).

The recordings for each trial were transformed to a time–frequency representation using a Morlet wavelet transform before the averaging procedure. Then, at least 100 time–frequency representations were averaged across trials, and averaged across 25 sensors again for each right and left hemisphere. This second analysis will reveal a mixture of evoked and induced power changes in each hemisphere because phase stability in the responses is not necessary. This Morlet wavelet transform was applied to the 20–100 Hz frequency range of the MEG epochs. Wavelet center frequencies were 20, 24.2, 28.4, 32.6, 36.8, 41.1, 45.3, 49.5, 53.7, 57.9, 62.1, 66.3, 70.5, 74.7, 79.0, 83.2, 87.4, 91.6, 95.8, and 100 Hz. Baseline levels were subtracted from each time–frequency map (-50 to 0 ms relative to stimulus onset). All spectral analyses were performed using an analyzer (Brain Vision; Brain Product, GmbH).

### Anatomical investigation

All participants underwent T1-weighted MRI examination (Signa Excite HD 1.5 T system; GE Yokogawa). To superpose the coordinate system of MEG on the MRI images, T1-weighted MRI was performed with spherical lipid markers placed at the five MEG fiduciary points. These MRI images consisted of 166 sequential horizontal slices of 1.2 mm thickness, with resolution of 512 × 512 points in a 261 × 261 mm field of view. After reconstructing three-dimensional MRI images, the best-fit sphere was determined for each participant’s head.

### Statistics

Statistical significance of the effects was inferred using un-paired *t* tests. Pearson’s product moment correlation coefficient (*r*) was used for correlation analyses.

## Results

### Dipole location estimated by the equivalent current dipole (ECD) model

Fig 2 presents results of magnetic responses following AM stimuli obtained from a representative, control participant. The AM-evoked response culminated at around 100 ms after the onset of AM stimulation (Fig 2A). As shown in Fig 2B, the ECD for AM-evoked MEG response was in the occipital association area. Similarly to the findings shown for this participant, during AM stimuli, 17 of 34 participants (8 of 17 healthy participants, 9 of 17 AD patients) showed magnetic responses by which we were able to estimate dipoles that met the acceptable criteria in the cortical region with the single ECD model. These estimated ECDs were identified in the right hemisphere in 15 participants (8 of 8 healthy participants, 7 of 9 AD patients) or in the left hemisphere in two participants (2 of 9 AD patients). The mean moment of estimated ECD was 7.8 ± 3.8 nAm (mean ± SD, *n* = 8) for healthy participants, whereas that for AD patients was 7.1 ± 4.8 nAm (mean ± SD, *n* = 9). The estimated dipole latencies or moments did not differ significantly between AD patients and healthy participants (latencies, *t* = 0.09, *p* > 0.05; dipole moments, *t* = 0.33, *p* > 0.05).

However, 17 of the 34 participants (9 of 17 healthy participants, 8 of 17 AD patients) showed magnetic fields with a complex pattern consisting of multiple flux-out or flux-in foci. Therefore, the estimated ECDs in these cases did not fulfill the acceptable criteria for additional single ECD analysis.

### Stimulus-induced changes in gamma band (60–80 Hz)

**Fig 4:**
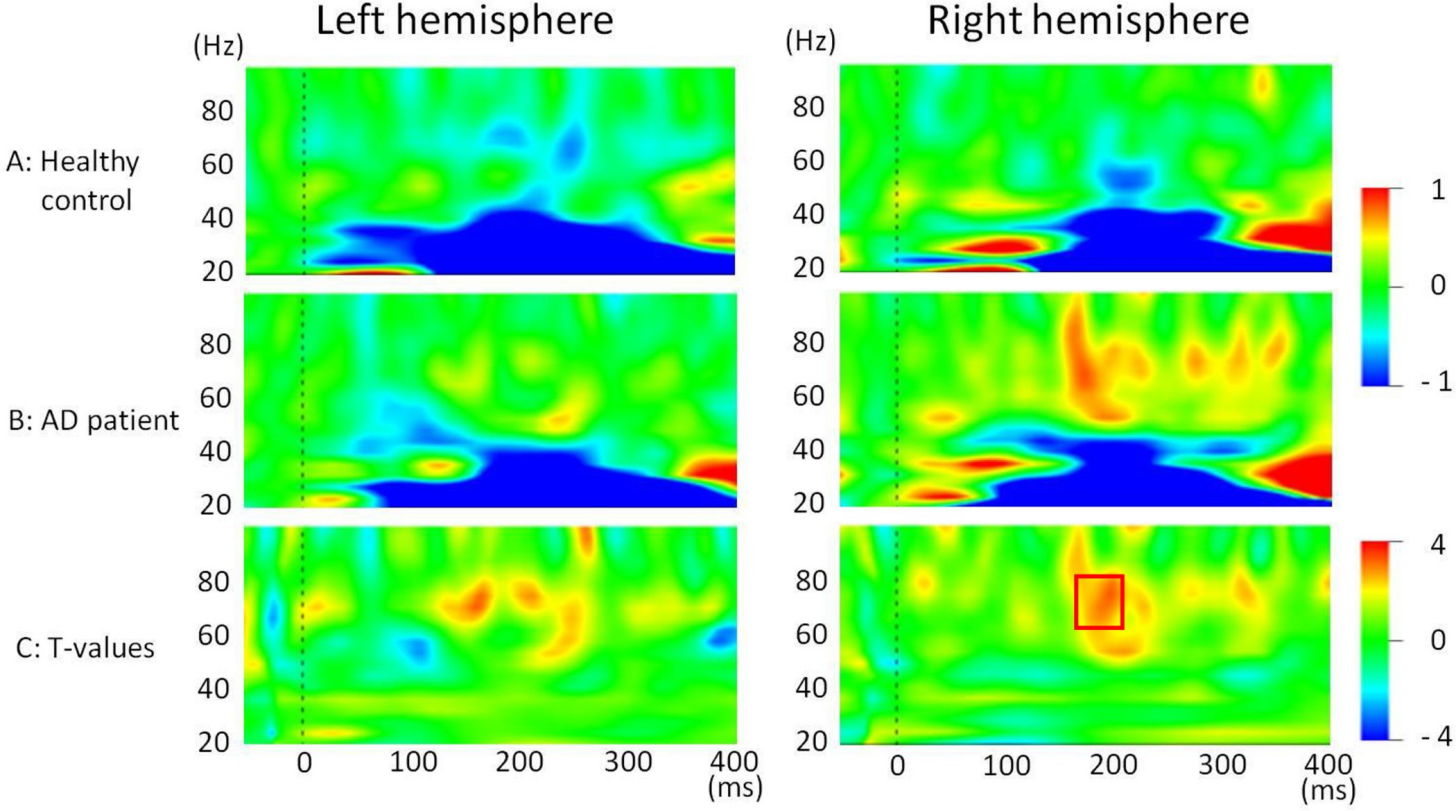
Grand average stimulus-related time–frequency maps using a Morlet wavelet transform for (A) healthy controls (*n* = 17) and (B) AD patients (*n* = 17) for both hemispheres. This analysis will reveal a mixture of evoked and induced power changes because phase stability in the responses is not necessary. (C) Stimulus-related time–frequency T-value maps for healthy control and Alzheimer’s disease participants.

**Fig 5:**
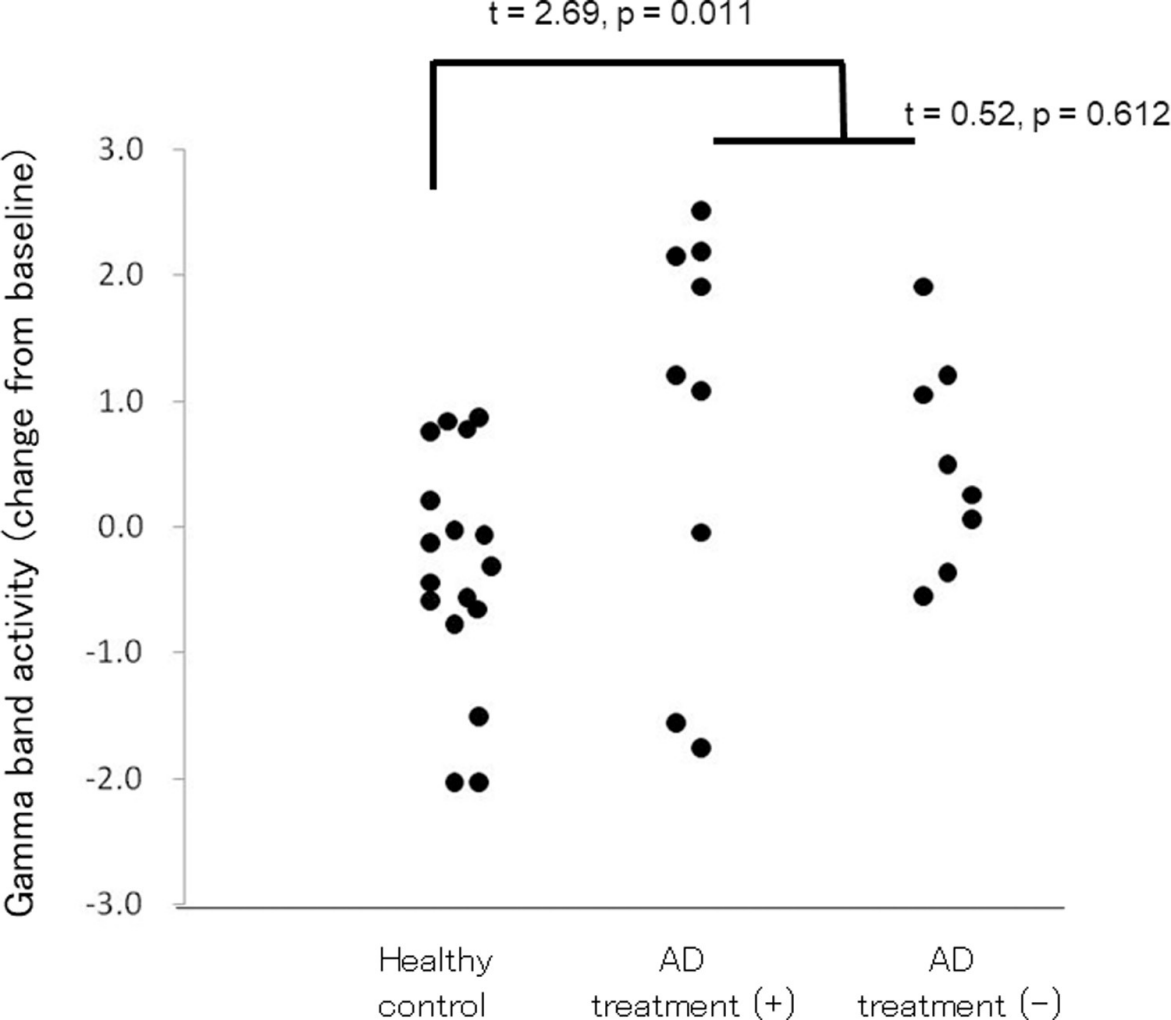
Changes in gamma band activity (positive value means increase, 62.11–70.53 Hz, 170–200 ms after stimulus). “AD treatment (+)” denotes patients treated with 5 mg of donepezil hydrochloride. “AD treatment (-)” denotes AD patients with no drug treatment. Increases in gamma band activity in AD patient were greater than those in the control group (*t* = 2.69, *p* = 0.011), although drug treatment has no significant effect on these changes (*t* = 0.52, *p* = 0.612).

As shown in Fig 4B, a time–frequency representation demonstrated that AM stimuli elicited a robust increase in the gamma band (50–80 Hz) in right hemispheres in AD patients. Unpaired *t*-tests comparing findings for the two groups demonstrated a prominent gamma (60–80 Hz) increase of about 170 ms after stimuli in the right hemisphere in the AD group (Fig 4C). To assess effects of treatment with 5 mg of donepezil hydrochloride on this robust difference 170–200 ms after stimuli, we calculated the average value of the wavelet coefficients in the right hemisphere (62.1–70.5 Hz, 170–200 ms after stimulus) for healthy controls, AD patients with and without drug treatment (Fig 5). As shown in Fig 5, no robust effect of treatment with 5 mg of donepezil hydrochloride was apparent from this measurement (*t* = 0.52, *p* = 0.612).

### Memory performance correlations in AD patients

**Fig 6:**
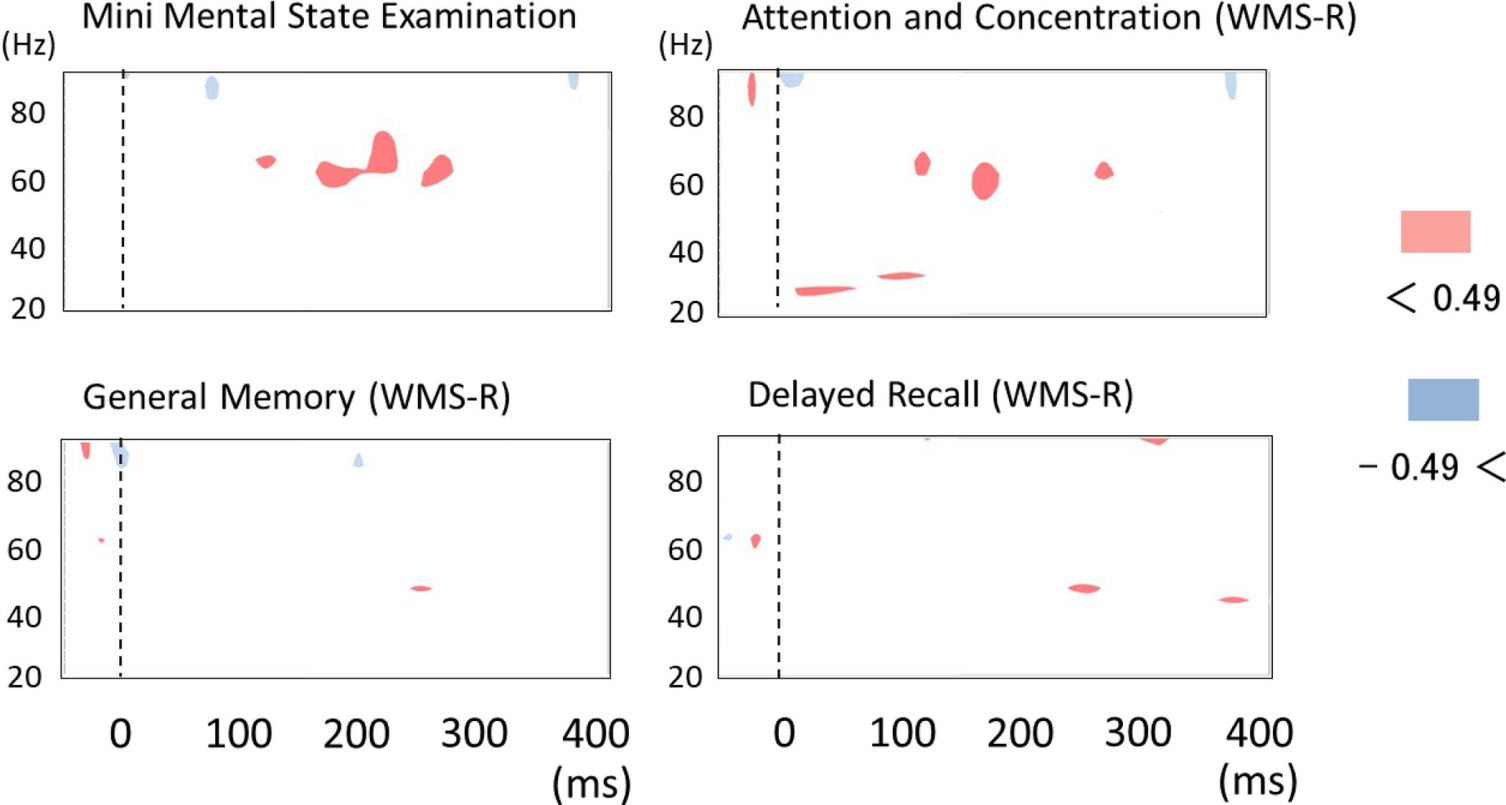
Time–frequency maps of correlation coefficient between visual stimulation induced power change in right hemisphere and Mini Mental State Examination (MMSE) score or Wechsler Memory Scale-Revised (WMS-R) indexes in AD patients (*n* = 17). Red areas show positive correlation (correlation coefficient > 0.5). Blue areas show negative correlation (correlation coefficient < - 0.5). Prominent positive correlation is found for the MMSE score and WMS-R index for attention and concentration at around 200 ms after stimuli.

Because of significant differences in AM-induced gamma band changes between AD patients and healthy controls in the right hemisphere, we investigated the relation between AM-induced oscillation in the right hemisphere and memory performance. We made time–frequency maps of correlation coefficients between visual-stimulation-induced power changes in the right hemisphere and MMSE scores or WMS-R indexes in AD patients (Fig 6). As shown in Fig 6 (upper maps), prominent positive correlation was found between gamma band synchronization (around 200 ms after AM stimuli) and the MMSE total score or one of the WMS-R index (attention and concentration).

## Discussion

The present study demonstrated that 8 of 17 healthy participants (47.1%) had a plausible single ECD source in posterior regions. Using similar AM stimuli, earlier studies of healthy participants demonstrated that 9 out of 12 participants (75.0%) had magnetic responses that could be used to estimate the cortical origins with the single ECD model, and demonstrated that these estimated cortical origins corresponded anatomically to the middle temporal area, third visual cortex accessory, and posterior end of superior temporal sulcus [13]. These locations were identical to those found not only from an earlier MEG study [22–24], but also from PET and fMRI studies [25, 26], which measured regional activation during visual motion stimuli. For the present study, to elucidate neural substrates in conjunction with impaired visual motion processing in AD patients, we studied ECD sources in AD patients and detected plausible single ECD sources in posterior regions in 9 of 17 AD patients (52.9%). This report is the first of a study of AM-evoked MEG potentials in AD patients. However, we failed to find different characteristics of ECD sources between the AD and control groups. The dipole strength or peak latency did not differ between the two groups. We were unable to draw any definitive conclusions because of the small sample size. Further investigation with a larger sample must be done to ascertain whether differences exist between normal elderly and AD patients in visually motion-evoked, extracellular current flow associated with massively summed postsynaptic potentials.

From this MEG study, we obtained time–frequency maps averaged over each trial of AM stimulation after Morlet wavelet transformation. Consistent with our hypothesis, we found elevated induced gamma-band (60–80 Hz) activity in the AD group. Although the finding of gamma band activity in AD patients remains controversial, this report is the first of a study showing that AM stimuli induce the augmented activity in the gamma-band (60–80 Hz) in early-stage AD patients. Using a considerably different analytical method (i.e., spontaneous EEG oscillations), one earlier study demonstrated elevated gamma band EEG power in AD patients, which was positively correlated with cognitive performance [12]. This earlier study was unable to demonstrate the precise time course of gamma band activity because of the methodological difference (i.e. they calculate the average power over a long interval). However, consistent with our current results, this earlier frequency domain approach demonstrated the possibility that cortical activities are preserved or that they are rather higher in AD. In contrast to our current findings, one earlier study demonstrated decreased gamma band power in a 40-Hz steady-state response (SSR) paradigm in AD [27]. This result cannot be compared directly with our current results because the generative mechanism of SSR differs from that of induced gamma band activities. Actually, SSR is a sinusoidal response at the driving stimulus frequency. It is elicited by simple auditory stimuli, which can be interpreted as a natural resonance frequency of the brain and which can be related to primary sensory processing [28].

Lower synchronization of the global field gamma band oscillation in AD has been reported from several earlier studies [29, 30]. This lower global field synchronization in the gamma band is explainable by the loss of long distance cortico-cortical connections that characterize AD because synchronization is a numerical property of the association between two or more sensors. These results are consistent with the concept that gamma-band oscillation between the neural networks is necessary for cortical information processing [31]. Our currently obtained results do not contradict earlier results because the localized gamma band activity recorded in each sensor can be elevated, reflecting the compensation mechanism for decreased long-distance connectivity.

Gamma band activity has been thought to account for inhibitory–excitatory neuronal dynamic alteration in terms of postsynaptic potentials in synchronously activated pyramidal cells. It is well established that inhibitory interneuron networks play a prominent role in the generation of gamma-band activity [32, 33]. The activation of these interneuron networks is attributable to excitatory drive from pyramidal cells [10]. Although it remains unclear which pathological mechanisms facilitate gamma band oscillations in AD, over-activation of both inhibitory and excitatory neuron in AD patients seem to account for elevated gamma band oscillations. In fact, recent reports have described that neuronal dysfunction in AD is associated with excitotoxic neurodegeneration [1–4]. Subsequent development of compensatory inhibitory mechanisms also contributes to pathology in AD patients [7]. Results of a transgenic mice study indicate this compensatory inhibitory mechanism in the hippocampus [8]. These findings support the over-activation of both inhibitory and excitatory neuron in AD patients, and also suggest that the compensating up-regulation of inhibitory neurons contributes to neuronal resistance to the excite-toxic disease process in AD. In our current study, AD patients who had higher gamma band activity after AM stimuli demonstrated higher performance of MMSE and of a WMS-R index for attention and concentration. This positive correlation might reflect the compensating mechanism of inhibitory neurons, which preserve pyramidal neurons from excitotoxicity in the posterior association area.

Generally, event-related gamma-band activity has been thought to reflect ongoing cognitive processes [11, 34–40]. Especially, the attention seems to play an important role in gamma oscillations. Sokolov et al. (1999) demonstrated that the effects of attention to the stimuli are specific to the gamma-band MEG activity after visual motion stimulation. The question arises of whether elevated gamma band activity in AD patients found in our current study reflects their deviated visual attention to the moving stimuli. Although all participants in our current study were instructed to devote their attention to a green spot as a static fixation point, AD patients might have allocated their attention to a moving bar instead of a fixation point because of their distractibility by moving visual stimuli [41]. However, this assumption cannot explain our results straightforwardly. Our study showed that AD patients who had higher gamma band activity tend to show less severe deficit in cognitive performance, and accordingly seem to be less likely to be distracted by moving visual stimuli. Further investigations using other visual tasks that require various degrees of attentional effort and using eye-gaze tracking systems must be undertaken to draw definitive conclusions.

Abundant gamma activities are apparently specific to the early stage of AD because compensative inhibitory mechanisms seem to operate at the onset of AD pathology, whereas loss of such mechanisms might facilitate or be caused by further progression of AD pathology [7]. In addition, sparseness of neuronal networks in severe AD must result in a decrease in neural oscillations. For better understanding of the underlying mechanisms which contribute to the excess gamma-band activity in early-stage AD patients, additional studies must be conducted using various cognitive tasks including AD patients with various degrees of disease severity. Results of our current study suggest the great possibility of these gamma band analyses, at least for early diagnosis of AD.

This study has some limitations. First, we did not employ positron emission tomography imaging or cerebrospinal fluid tests to ascertain whether amyloid beta and tau pathology are present in the brain. Secondly, further studies with various types of dementia must be conducted to ascertain conclusively whether the MEG findings in this study are specific for AD. Thirdly, this study is a sensor-level analysis. Further study with source-level analysis for the gamma band oscillations might enable us to identify brain regions that are specific for the early diagnosis of AD.

## Supporting information

S1 File (ZIP)
